# Editorial: Bone Marrow T Cells at the Center Stage in Immunological Memory

**DOI:** 10.3389/fimmu.2016.00596

**Published:** 2016-12-14

**Authors:** Francesca Di Rosa, Tania H. Watts

**Affiliations:** ^1^Institute of Molecular Biology and Pathology, Consiglio Nazionale delle Ricerche, c/o Department of Molecular Medicine, Sapienza University, Rome, Italy; ^2^Department of Immunology, University of Toronto, Toronto, ON, Canada

**Keywords:** T cells, bone marrow, immunological memory, bone and bones, hematopoiesis, cancer

**Editorial on the Research Topic**

**Bone Marrow T Cells at the Center Stage in Immunological Memory**

The notion that bone marrow (BM) T cells give a key contribution to adaptive immunity is increasingly recognized (1–3). Researchers now more often include the BM when analyzing T cell responses in experimental mouse models (4, 5) or when providing an overview of memory T cell compartmentalization (6). Translation of BM T cell knowledge into medicine has begun. Promising results of the first clinical trial using BM T cells in the treatment of multiple myeloma (MM) were reported last year (7). Further applications are expected in the near future, as BM T cells have been involved in a variety of processes, going from normal hematopoiesis to bone resorption in patients affected by hyperparathyroidism (8, 9).

This research topic on BM T cells contains two sections. The first one contains original research contributions on BM memory CD4 and CD8 T cells in mouse models (Hojyo et al.; Geerman et al.) and hosts a debate on the role of BM memory T cells in systemic or localized memory (Di Rosa; Sercan-Alp and Radbruch; Di Rosa and Gebhardt). In the second one, emerging scenarios in translational medicine in different fields (e.g., hematology, oncology, transplantation immunology, osteoimmunology, etc.) are discussed (Wakkach et al.; Borrello and Noonan; Szyska and Na; Pacifici; Bonomo et al.).

## Memory T Cells in the BM

The BM harbors a high frequency of antigen-specific memory T cells against vaccines, pathogens, and tumors and is considered a major site for the maintenance of memory T cells (reviewed by Di Rosa and Gebhardt). In addition to conventional memory T cells, another class of non-recirculating subsets—the so-called tissue-resident memory T cells (Trm)—has recently been identified in several non-lymphoid organs including skin, gut, and brain (10, 11). These cells, which can provide a first-line defense against reinfection at barrier surfaces, are characterized by expression of CD69 as well as integrins such as CD103 and VLA1, which can contribute to their tissue retention (reviewed in Di Rosa and Gebhardt). BM has a high proportion of CD69^+^ memory T cells (2, 3, 12), as confirmed in an original report by Geerman et al. in this research topic. However, the expression of CD69 may not be sufficient to define these T cells as “tissue resident.” Di Rosa and Gebhardt discuss the evidence that BM T cells are largely circulatory, likely stopping over temporarily in BM niches where they receive survival signals, before re-entering the circulation.

An issue of some debate has been the extent of homeostatic proliferation of the memory T cells in these niches [Di Rosa; Sercan-Alp and Radbruch; (3, 13–16)]. Sercan-Alp and Radbruch have suggested (3) that the level of homeostatic proliferation measured by BrdU is overestimated. However, this remains a point of contention. As often found when research groups disagree, the experimental details may offer a solution. One group found, for example, that MyD88 negative mice did not have unexpectedly high rates of BrdU incorporation (Sercan-Alp and Radbruch), suggesting that the BrdU may have been LPS contaminated. Another found that proliferation rates were similar with BrdU and CFSE labels (13). As documented by Di Rosa in her commentary, a variety of experimental approaches have provided evidence that the level of proliferation of memory T cells in the BM, while low, is higher than the level of homeostatic proliferation of T cells in spleen or LN. Thus, it is likely that the niches in the BM that are rich in cytokines such as IL-7 and IL-15, while largely providing survival signals may also induce a low level of proliferation, sufficient to at least partially support homeostasis. A recent hypothesis proposes that memory T cells circulating through the BM may stop to rest for a while in dedicated niches supporting quiescence and/or proliferate in distinct niches for self-renewal, before moving on (16).

In an original research article, Geerman et al. provide evidence that the frequency and phenotype of different subsets of memory T cells as well as their expression of cytokine receptors was similar in different bones in the steady state and after an acute systemic infection with lymphocytic choriomeningitis. This is reassuring for investigators who may wish to use different bones in their studies. Of note, the vertebrae, which contain the most BM cells, also provide the most abundant source of T cells.

In an original research contribution, Hojyo et al. focus on memory CD4 T cells and show that B cell depletion increases the number of CD49b^+^Tbet^+^ TCR transgenic CD4 memory T cells in the BM. Whether B cell depletion has a direct effect on the CD4 T cells or affects their access to another factor which in turn regulates their expression of CD49^+^ and/or BM localization is not yet clear.

## BM T Cells in Translational Medicine

The activation state of freshly isolated BM T cells, e.g., resulting from exposure to IL-15 in the organ, together with their prompt response to *in vitro* stimulation makes these cells ideal candidates for adoptive transfers in conditions requiring highly active effectors (17–19). The article by Borrello and Noonan recapitulates concepts and results on the use of marrow-infiltrating lymphocytes (MILs) against MM in humans and discusses the unique opportunity to exploit BM T cells in adoptive T-cell therapy against both hematological and solid cancers. Moreover, MIL transfer might ameliorate bone disease in MM patients, by switching BM T cells from Th17 to Th1 [Borrello and Noonan; (20)].

By contrast, in HSC transplantation (HSCT), donor T cell effector function against host BM stroma is detrimental for donor HSC seeding and hematopoiesis reconstitution. Starting with the recent recognition that BM is a major target organ in GVHD after allogeneic HSCT in leukemic patients (21), Szyska and Na discuss some possible mechanisms underlying this adverse effect, e.g., T-cell-derived cytolytic factors and cytokines can damage osteoblasts, endothelia, and surrounding cells, while replenishment of destroyed niches by hematopoietic cells is impaired.

Two articles link BM T cell-derived TNF-alpha and IL-17 to altered bone metabolism in human diseases. Pacifici discusses the evidence suggesting that catabolic effects of parathyroid hormone on bone in patients affected by hyperparathyroidism relies on Th17 cell-induced RANKL release by osteoblasts and osteocytes, with subsequent osteoclast-mediated bone resorption (9). Wakkach et al. give an overview of the mechanisms supporting bone destruction in inflammatory bowel disease and propose that TNF-alpha-producing Th17 cells in the BM sustain bone loss in patients with Crohn’s disease (22).

Bonomo et al. review the evidence that BM T cells are at the cross-roads between immunity, bone metabolism, and hematopoiesis and propose that T cells act as messengers who “bring the news” from the periphery to the BM. According to this view, activated T cells enter the BM and modulate BM-resident cell function, ultimately tuning blood cell production and bone remodeling to the class of peripheral immune response (Bonomo et al.).

## Author Contributions

TW wrote the paragraph on memory T cells in the BM; FD wrote the paragraph on BM T cells in Translational Medicine; FD and TW together wrote the remaining parts and edited the final text.

## Conflict of Interest Statement

The authors declare that the research was conducted in the absence of any commercial or financial relationships that could be construed as a potential conflict of interest.

## References

[1] Di Rosa F. T-lymphocyte interaction with stromal, bone and hematopoietic cells in the bone marrow. Immunol Cell Biol (2009) 87:20–9. 10.1038/icb.2008.84 19030018

[2] Okhrimenko A Grun JR Westendorf K Fang Z Reinke S von Roth P Human memory T cells from the bone marrow are resting and maintain long-lasting systemic memory. Proc Natl Acad Sci U S A (2014) 111:9229–34. 10.1073/pnas.1318731111 24927527 PMC4078840

[3] Sercan Alp O Durlanik S Schulz D McGrath M Grun JR Bardua M Memory CD8(+) T cells colocalize with IL-7(+) stromal cells in bone marrow and rest in terms of proliferation and transcription. Eur J Immunol (2015) 45:975–87. 10.1002/eji.201445295 25639669 PMC4415462

[4] Bolinger B Sims S Swadling L O’Hara G de Lara C Baban D Adenoviral vector vaccination induces a conserved program of CD8(+) T cell memory differentiation in mouse and man. Cell Rep (2015) 13:1578–88. 10.1016/j.celrep.2015.10.034 26586434 PMC4670868

[5] Jung YW Kim HG Perry CJ Kaech SM. CCR7 expression alters memory CD8 T-cell homeostasis by regulating occupancy in IL-7- and IL-15-dependent niches. Proc Natl Acad Sci U S A (2016) 113:8278–83. 10.1073/pnas.1602899113 27385825 PMC4961181

[6] Farber DL Yudanin NA Restifo NP. Human memory T cells: generation, compartmentalization and homeostasis. Nat Rev Immunol (2014) 14:24–35. 10.1038/nri3567 24336101 PMC4032067

[7] Noonan KA Huff CA Davis J Lemas MV Fiorino S Bitzan J Adoptive transfer of activated marrow-infiltrating lymphocytes induces measurable antitumor immunity in the bone marrow in multiple myeloma. Sci Transl Med (2015) 7:288ra78. 10.1126/scitranslmed.aaa7014 25995224 PMC4634889

[8] Kim S Park K Choi J Jang E Paik DJ Seong RH Foxp3+ regulatory T cells ensure B lymphopoiesis by inhibiting the granulopoietic activity of effector T cells in mouse bone marrow. Eur J Immunol (2015) 45:167–79. 10.1002/eji.201444532 25348202

[9] Li JY D’Amelio P Robinson J Walker LD Vaccaro C Luo T IL-17A is increased in humans with primary hyperparathyroidism and mediates PTH-induced bone loss in mice. Cell Metab (2015) 22:799–810. 10.1016/j.cmet.2015.09.012 26456334 PMC4635034

[10] Schenkel JM Masopust D. Tissue-resident memory T cells. Immunity (2014) 41:886–97. 10.1016/j.immuni.2014.12.007 25526304 PMC4276131

[11] Mueller SN Mackay LK. Tissue-resident memory T cells: local specialists in immune defence. Nat Rev Immunol (2016) 16:79–89. 10.1038/nri.2015.3 26688350

[12] Shinoda K Tokoyoda K Hanazawa A Hayashizaki K Zehentmeier S Hosokawa H Type II membrane protein CD69 regulates the formation of resting T-helper memory. Proc Natl Acad Sci U S A (2012) 109:7409–14. 10.1073/pnas.1118539109 22474373 PMC3358871

[13] Parretta E Cassese G Santoni A Guardiola J Vecchio A Di Rosa F Kinetics of in vivo proliferation and death of memory and naive CD8 T cells: parameter estimation based on 5-bromo-2’-deoxyuridine incorporation in spleen, lymph nodes, and bone marrow. J Immunol (2008) 180:7230–9. 10.4049/jimmunol.180.11.7230 18490722

[14] Di Rosa F Maintenance of memory T cells in the bone marrow: survival or homeostatic proliferation? Nat Rev Immunol (2016) 16:271 10.1038/nri.2016.31 26996200

[15] Sercan-Alp O Radbruch A The lifestyle of memory CD8(+) T cells. Nat Rev Immunol (2016) 16:271 10.1038/nri.2016.32 26996198

[16] Di Rosa F. Two niches in the bone marrow: a hypothesis on life-long T cell memory. Trends Immunol (2016) 37:503–12. 10.1016/j.it.2016.05.004 27395354

[17] Di Rosa F Santoni A. Bone marrow CD8 T cells are in a different activation state than those in lymphoid periphery. Eur J Immunol (2002) 32:1873–80. 10.1002/1521-4141(200207)32:7<1873::AID-IMMU1873>3.0.CO;2-P 12115606

[18] Quinci AC Vitale S Parretta E Soriani A Iannitto ML Cippitelli M IL-15 inhibits IL-7Ralpha expression by memory-phenotype CD8(+) T cells in the bone marrow. Eur J Immunol (2012) 42:1129–39. 10.1002/eji.201142019 22539288

[19] Snell LM Lin GH Watts TH. IL-15-dependent upregulation of GITR on CD8 memory phenotype T cells in the bone marrow relative to spleen and lymph node suggests the bone marrow as a site of superior bioavailability of IL-15. J Immunol (2012) 188:5915–23. 10.4049/jimmunol.1103270 22581858

[20] Noonan K Marchionni L Anderson J Pardoll D Roodman GD Borrello I. A novel role of IL-17-producing lymphocytes in mediating lytic bone disease in multiple myeloma. Blood (2010) 116:3554–63. 10.1182/blood-2010-05-283895 20664052 PMC4017298

[21] Mensen A Johrens K Anagnostopoulos I Demski S Oey M Stroux A Bone marrow T-cell infiltration during acute GVHD is associated with delayed B-cell recovery and function after HSCT. Blood (2014) 124:963–72. 10.1182/blood-2013-11-539031 24833353

[22] Ciucci T Ibanez L Boucoiran A Birgy-Barelli E Pene J Abou-Ezzi G Bone marrow Th17 TNFalpha cells induce osteoclast differentiation, and link bone destruction to IBD. Gut (2015) 64:1072–81. 10.1136/gutjnl-2014-306947 25298539

